# High-confidence placement of low-occupancy fragments into electron density using the anomalous signal of sulfur and halogen atoms

**DOI:** 10.1107/S2059798324004480

**Published:** 2024-06-05

**Authors:** Shumeng Ma, Shymaa Damfo, Matthew W. Bowler, Vitaliy Mykhaylyk, Frank Kozielski

**Affiliations:** a School of Pharmacy, University College London, 29-39 Brunswick Square, London WC1N 1AX, United Kingdom; b Pharmacognosy and Pharmaceutical Chemistry Department, College of Pharmacy, Taibah University, Al-Madinah Al-Mounawarah 30078, Saudi Arabia; c European Molecular Biology Laboratory, 38000 Grenoble, France; d Diamond Light Source, Harwell Science and Innovation Campus, Chilton, Didcot OX11 0DE, United Kingdom; University of Oxford, United Kingdom

**Keywords:** SARS-CoV-2 nsp1, fragment-based drug discovery, fragment orientations, anomalous scattering, *PanDDA*, halogens, COVID-19, low occupancy, radiation damage

## Abstract

Fragment-based drug design using X-ray crystallography is a powerful technique to enable the development of new lead compounds, or probe molecules, against biological targets. This study addresses the need to determine fragment binding orientations for low-occupancy fragments with incomplete electron density, an essential step before further development of the molecule. Halogen atoms play multiple roles in drug discovery due to their unique combination of electronegativity, steric effects and hydrophobic properties. Fragments incorporating halogen atoms serve as promising starting points in hit-to-lead development as they often establish halogen bonds with target proteins, potentially enhancing binding affinity and selectivity, as well as counteracting drug resistance. Here, the aim was to unambiguously identify the binding orientations of fragment hits for SARS-CoV-2 nonstructural protein 1 (nsp1) which contain a combination of sulfur and/or chlorine, bromine and iodine substituents. The binding orientations of carefully selected nsp1 analogue hits were focused on by employing their anomalous scattering combined with *Pan-Dataset Density Analysis* (*PanDDA*). Anomalous difference Fourier maps derived from the diffraction data collected at both standard and long-wavelength X-rays were compared. The discrepancies observed in the maps of iodine-containing fragments collected at different energies were attributed to site-specific radiation-damage stemming from the strong X-ray absorption of I atoms, which is likely to cause cleavage of the C—I bond. A reliable and effective data-collection strategy to unambiguously determine the binding orientations of low-occupancy fragments containing sulfur and/or halogen atoms while mitigating radiation damage is presented.

## Abstract 2

Anomalous scattering and *PanDDA* were employed to determine the binding orientation(s) of novel planar and low-occupancy fragment hits containing sulfur and/or halogen atoms that bind to SARS-CoV-2 nsp1. This approach was validated for these challenging-to-fit fragments with an anomalous data-collection strategy designed to mitigate radiation-damage effects, which are known to particularly affect halogenated fragments due to their higher X-ray absorption cross sections.

## 1. Introduction

In fragment-based drug discovery (FBDD), the use of small molecular fragments, typically with a molecular weight of less than 300 Da, offers distinct advantages. Due to their small size and low chemical complexity, fragments tend to yield a higher hit rate, efficiently cover a wider range of target binding sites and provide flexibility for subsequent structural modifications (Zimmermann *et al.*, 2014 ▸; Baker, 2013 ▸). These attributes make fragment hits excellent starting points for drug development. High-throughput screening (HTS) methods are employed in FBDD to efficiently identify fragment hits with the potential to be developed into lead compounds. Among these HTS methods, X-ray crystallography is considered to be an indispensable technique due to its exceptional ability to elucidate the three-dimensional structures of fragment–protein complexes in addition to hit identification, which in turn empowers the rational design and optimization of lead compounds (Hartshorn *et al.*, 2005 ▸). However, due to the features of low binding affinity, the co-existence of alternative binding orientations and low occupancy in binding sites, fragments tend to display incomplete electron density in the electron-density maps. In addition, due to their low molecular weight, highlighted within the ‘rule of three’ (RO3; Congreve *et al.*, 2003 ▸), and the incorporation of at least one ring (Giordanetto *et al.*, 2019 ▸), a large proportion of fragments are planar or quasi-symmetric. These factors render fragment fitting into incomplete density and determination of their binding orientation(s) challenging. However, this problem can be overcome by using fragments incorporating halogen atoms. The distinctive combination of electronegativity, steric effects and hydrophobic properties allows halogen atoms to intricately modulate crucial aspects of drug binding, such as potency, metabolic stability, lipophilicity and permeability (Hernandes *et al.*, 2010 ▸; Wang *et al.*, 2022 ▸). Over the past decade, halogenated ligands have received increased attention because of their exceptional attributes, including enhanced binding affinity and selectivity for targets, and the potential to counteract drug resistance imparted by the formation of a noncovalent interaction with target molecules, called the ‘halogen bond’ (Baumli *et al.*, 2010 ▸; Hardegger *et al.*, 2011 ▸). Various halogenated fragment libraries, such as the Halo Library, FragLites and HEFLib (Chopra *et al.*, 2023 ▸; Heidrich *et al.*, 2019 ▸; Wood *et al.*, 2019 ▸), have been designed to probe the halogen-bond interaction in macromolecules of interest to speed up the drug-discovery process.

In addition to these attributes that are beneficial in interactions with proteins, halogens also have a significant anomalous signal at wavelengths commonly used at synchrotron beamlines. It is common to collect anomalous diffraction data for the identification and orientation of halogenated small molecules in the binding sites of macromolecules (Coleman *et al.*, 2020 ▸; Pflug *et al.*, 2012 ▸). However, for halogenated fragments this approach has only been applied in a limited number of studies. For example, in hot-spot identification studies the anomalous signal from bound halogenated fragments has been used to locate the binding pockets of HIV reverse transcriptase (Bauman *et al.*, 2016 ▸; Chopra *et al.*, 2023 ▸), HIV protease (Tiefenbrunn *et al.*, 2014 ▸) and *Thermus thermophilus* EF-Tu (Grøftehauge *et al.*, 2013 ▸). Other examples include cyclin-dependent kinase 2 (Wood *et al.*, 2019 ▸) and glycerol-3-phosphate dehydrogenase (Choe *et al.*, 2002 ▸). In these studies, the anomalous signal from halogen atoms was used to confirm the binding and the binding orientations of fragments with reasonable electron density. This raises an important question about the feasibility of this approach when the fragment density is incomplete, as is commonly observed for low-occupancy fragments. Additionally, most of these anomalous data were collected at wavelengths between 0.9 and 1.8 Å (Bauman *et al.*, 2016 ▸; Blaney *et al.*, 2006 ▸; Choe *et al.*, 2002 ▸; Tiefenbrunn *et al.*, 2014 ▸; Wood *et al.*, 2019 ▸; Grøftehauge *et al.*, 2013 ▸), where the protein crystals may experience site-specific radiation damage due to enhanced X-ray absorption above the *L* edge of iodine (5.2 keV) or the *K* edge of bromine (13.5 keV). If no account is taken of this phenomenon, it is possible that site-specific radiation damage could lead to a distortion of the peaks associated with iodine or bromine in the anomalous difference maps (Ravelli *et al.*, 2003 ▸). This distortion has the potential to complicate the process of fragment fitting. Therefore, it is necessary to consider the influence of radiation damage on the anomalous difference Fourier maps of iodo or bromo fragments (Zwart *et al.*, 2004 ▸) and to use a data-collection strategy that minimizes the effects of exposure to radiation.

*Pan-Dataset Density Analysis* (*PanDDA*) is a powerful tool for the identification of low-occupancy ligands that leverages ensemble refinement and multi-crystal averaging to enhance the accuracy of electron-density maps, revealing subtle differences that pinpoint the binding sites of fragment hits (Pearce, Bradley *et al.*, 2017 ▸; Pearce, Krojer *et al.*, 2017 ▸). It has been reported that the effectiveness of fragment binding detection by the inspection of anomalous difference Fourier density maps is superior (Wood *et al.*, 2019 ▸) or equal (Davison *et al.*, 2022 ▸) to that of *PanDDA*. Therefore, it is interesting to compare their effectiveness in the determination of binding orientations of low-occupancy fragments.

In this study, we describe our approach to the high-confidence placement of fragments binding to SARS-CoV-2 nsp1 that contain S and/or halogen atoms (Cl, Br and I) into electron density using anomalous diffraction. We chose SARS-CoV-2 nsp1 as a model system because its crystals routinely diffract to high resolution, fragments can easily be soaked in without compromising crystal quality and several hundred data sets are at our disposal. For our investigation, we selected fragments that had already displayed low occupancy and incomplete electron density. The effectiveness of anomalous difference Fourier maps and *PanDDA* in the placement of these low-occupancy fragments into incomplete electron density was investigated. Furthermore, a study was conducted to determine how site-specific radiation damage develops with X-ray absorption during the collection of diffraction data, exemplified by an iodine-containing fragment. Finally, we demonstrate that the integration of anomalous difference Fourier maps and *PanDDA* offers a reliable and effective strategy for fitting fragments into challenging electron density with a high degree of confidence.

## 2. Experimental

### 2.1. Expression, purification and crystallization of SARS-CoV-2 nsp1_10–126_

The N-terminal domain of SARS-CoV-2 nsp1_10–126_, containing residues 10 to 126 and named nsp1 throughout this manuscript, was expressed, purified and crystallized as described previously (Ma *et al.*, 2022 ▸). The crystallization condition used was Index screen (catalogue No. HR2-944-71; Hampton Research, Aliso Viejo, California, USA) condition 71 consisting of 0.1 *M* bis-Tris pH 6.5, 0.2 *M* NaCl, 25%(*w*/*v*) polyethylene glycol 3350.

Fragment hits 6A6, 1E7, 7G3, 9D4 and 11A7 were obtained from the Maybridge Ro3 library (Thermo Fisher Scientific, United Kingdom), while fragment hits 7H2_AL1, 7H2_AL2, 11A7_AL5 and 11A7_AL6 were purchased from Molport (Beacon, New York, USA), with a purity nominally exceeding 95%. The compounds were dissolved in DMSO-d_6_ (Sigma, St Louis, Missouri, USA; CAS No. 2206-27-1) at a concentration of 200 m*M*. Each stock (2 µl) was mixed with 8 µl crystallization condition, resulting in a fragment concentration of 40 m*M* in the crystallization condition, which also contained 20% DMSO-d_6_. Fragments or DMSO-d_6_ solutions (1.5 µl) were added to the approximately 1 µl volume of the crystallization drop, resulting in a final fragment concentration of 24 m*M* containing approximately 12% DMSO-d_6_. These drops were incubated at room temperature for 4–5 h. No additional cryoprotection was necessary as 25%(*w*/*v*) polyethylene glycol 3350 was present in the crystallization condition. The crystals were harvested using loops, cryocooled in liquid nitrogen and stored in pucks for sample shipment.

### 2.2. Data collection, structure determination and refinement

High-resolution data for nine nsp1–fragment complexes were collected on MASSIF-1 (Bowler *et al.*, 2015 ▸; Svensson *et al.*, 2015 ▸) at the European Synchrotron Radiation Facility (ESRF) at an incident beam energy *E_x_* of 12.8 keV with 360° of rotation and fine-slicing. The data were analysed in both the single-crystal and multi-crystal systems using *PanDDA*.

The long-wavelength diffraction experiments were carried out on beamline I23 at Diamond Light Source (DLS). The measurements were performed in a vacuum environment using the semi-cylindrical PILATUS 12M detector and multi-axis goniometer (Wagner *et al.*, 2016 ▸). During the measurements, the temperature of protein crystals mounted on copper sample holders was estimated to be ∼80 K. Each sample was exposed to X-rays at an incident beam energy of 4.5 keV. At this energy, the X-ray attenuation lengths of protein soaked with fragments containing sulfur, chlorine, bromine and iodine are practically the same (114, 114, 111 and 111 µm, respectively) and are very similar to that of protein alone (117 µm; values calculated at https://henke.lbl.gov/optical_constants/atten2.html). Therefore, when exposed to the same photon flux at 4.5 keV, samples with the same geometry (100 ×100 × 80 µm) absorb a very similar average dose per whole crystal as they are fully exposed, being smaller than the 200 × 200 µm X-ray beam. The dose was equal to 5.4 MGy per data set in this experiment, as calculated by *RADDOSE*-3*D* (Bury *et al.*, 2018 ▸). Consequently, the radiation damage induced in all fragments is expected to have the same dynamics and magnitude. This was essential in the interpretation of the results and their comparison, as exemplified in our studies of iodine-containing samples (7H2_AL2 and 11A7_AL5) which were also exposed at 5.3 and 9.0 keV (corresponding to wavelengths of 2.8, 2.3 and 1.4 Å, respectively).

For each sample, a 360° rotation data set with fine-slicing (0.10°) was collected to obtain high multiplicity, with the resolution limited only by the detector dimensions (1.8, 1.5 and 1.0 Å at wavelengths of 2.8, 2.3 and 1.4 Å, respectively). Data-collection statistics for data sets obtained on beamlines I23 and MASSIF-1 at wavelengths of 2.8 Å (4.5 keV) and 1.0 Å (12.8 keV), respectively, are summarized in Table 1 ▸.

The data-processing pipelines *fast_dp* (version 1.6.2), *xia*2 (version 3.12.0), *xia*2_*3dii* and *xia*2_*dials* (Winter *et al.*, 2022 ▸) were automatically employed. PDB entry 8a55 and the protein sequence were provided in ISPyB (Delagenière *et al.*, 2011 ▸) to trigger the *Dimple* processing pipeline (version 2.6.2; Wojdyr *et al.*, 2013 ▸) to generate anomalous difference Fourier maps, and *MrBUMP* (version 2.2.6; Keegan & Winn, 2008 ▸) was employed to find a molecular-replacement solution. Within the *Dimple* run, ten cycles of rigid-body refinement were performed, followed by four cycles of jelly-body refinement and eight cycles of restrained refinement, before starting to identify anomalous difference peaks (Wojdyr *et al.*, 2013 ▸).

**Figure 1:**
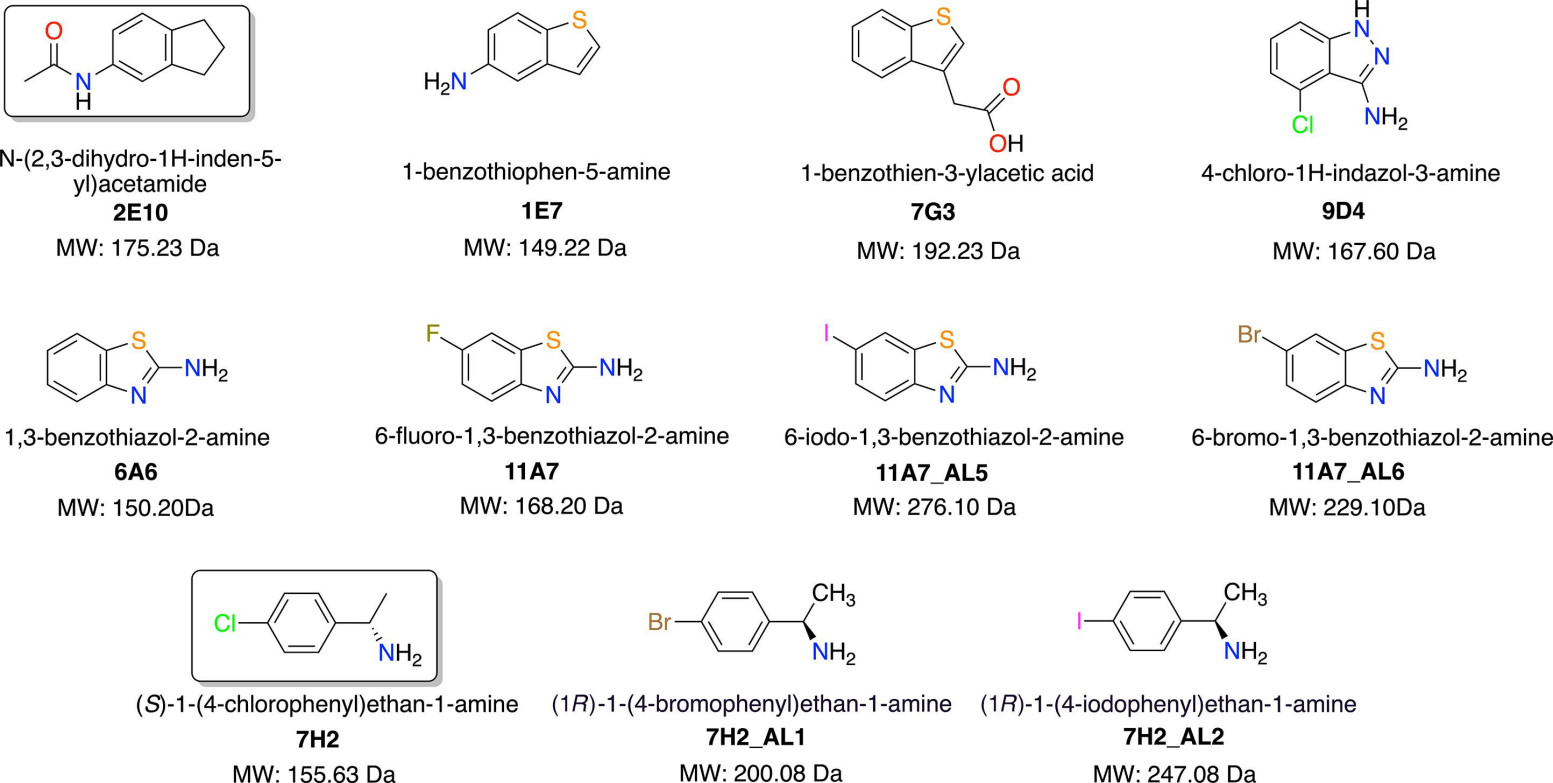
Chemical structures of fragment analogues containing S atoms and/or a chloro, bromo and iodo substituent that bind to nsp1. 2E10 and 7H2 (boxed) are two parental fragments that were reported in our previous publications (Borsatto *et al.*, 2022 ▸; Ma *et al.*, 2022 ▸).

To unambiguously identify the binding orientations of the fragment hits, the nsp1–fragment coordinates and anomalous difference Fourier maps were overlaid with 2*mF*_o_ − *DF*_c_ and *mF*_o_ − *DF*_c_ maps calculated from the high-resolution data (12.8 keV) for inspection in *Coot* (version 8.0; Emsley *et al.*, 2010 ▸). For 1E7, 6A6, 7G3, 9D4, 11A7, 11A7_AL5 and 11A7_AL6, nsp1 binding site A is located in proximity to Lys125, whereas for 7H2_AL1 and 7H2_AL2 the shallower binding site B is located adjacent to Pro109, as recently described (Ma *et al.*, 2022 ▸). Fragment hits were manually fitted into the fragment density, with S and/or halogen atoms positioned at the peak centres of the associated anomalous difference Fourier maps. Occupancy refinement was then conducted, which involved initially assigning a reasonable occupancy value to the fragments in *Coot* and then initiating occupancy refinement in *Phenix* (version 1.20.1-4487; Afonine *et al.*, 2012 ▸). The *mF*_o_ − *DF*_c_ maps around the fragments were then visually inspected, guiding the adjustment of the initial occupancy values. This was followed by iterative rounds of occupancy refinement in *Phenix* and further inspection in *Coot*, which continued until the electron density in the *mF*_o_ − *DF*_c_ maps around the fragments reached a minimum. Refinement statistics are shown in Table 2 ▸. Fig. 1 was prepared using *ChemDraw* (version 20.1; Cousins, 2005 ▸), whereas Supplementary Fig. S2, Fig. 3(*a*) and Figs. 4–6 were generated with *PyMOL* (version 2.4.1; Schrödinger).

### 2.3. PanDDA

Given the incomplete *mF*_o_ − *DF*_c_ maps that were observed for more than half of the fragment hits collected at 12.8 keV, *PanDDA* (version 0.2.14; Pearce, Bradley *et al.*, 2017 ▸) within *CCP*4 (version 7.1; Agirre *et al.*, 2023 ▸) was employed to determine whether more complete fragment density could be observed in event maps. To prepare for* PanDDA*, the coordinate and map files for each protein–fragment complex, together with the chemical structure files of the soaked fragments in PDB and CIF formats, were grouped into a single folder. The coordinate and map files for each complex were generated following MR by *Dimple* (Wojdyr *et al.*, 2013 ▸) in *CCP*4 (version 7.0.072; Agirre *et al.*, 2023 ▸) using PDB entry 8a55 as a search model (Ma *et al.*, 2022 ▸), while the chemical structure files for each fragment were generated by *eLBOW* in *Phenix* (version 1.19.1; Liebschner *et al.*, 2019 ▸). In addition, 40 high-resolution data sets of native nsp1 from the protein soaked in 12% DMSO were used in *PanDDA* to construct a ‘ground-state’ model of nsp1. To identify hits, *pandda.analyse* was run following the instructions at https://pandda.bitbucket.io/pandda/tutorials.html. Each interesting event was inspected with *pandda.inspect* through the *Coot* interface to confirm clear electron density for bound fragments. *PanDDA* maps of fragment density were captured and used for the preparation of Figs. 4, 5 and 6.

## 3. Results

Nine new nsp1-binding fragment analogues containing sulfur and/or halogen substituents were selected for this study. The chemical structures of the analogues and their parental fragment hits are shown in Fig. 1 ▸. The SMILES string computer-readable identifiers are provided in Supplementary Table S1. Except for 7H2, 7H2_AL1 and 7H2_AL2, which bind to binding site B, all other fragments were located in binding site A of nsp1 (Ma *et al.*, 2022 ▸).

### 3.1. Comparison of the quality of anomalous scattering data collected on MASSIF-1 and a dedicated long-wavelength beamline

**Figure 2:**
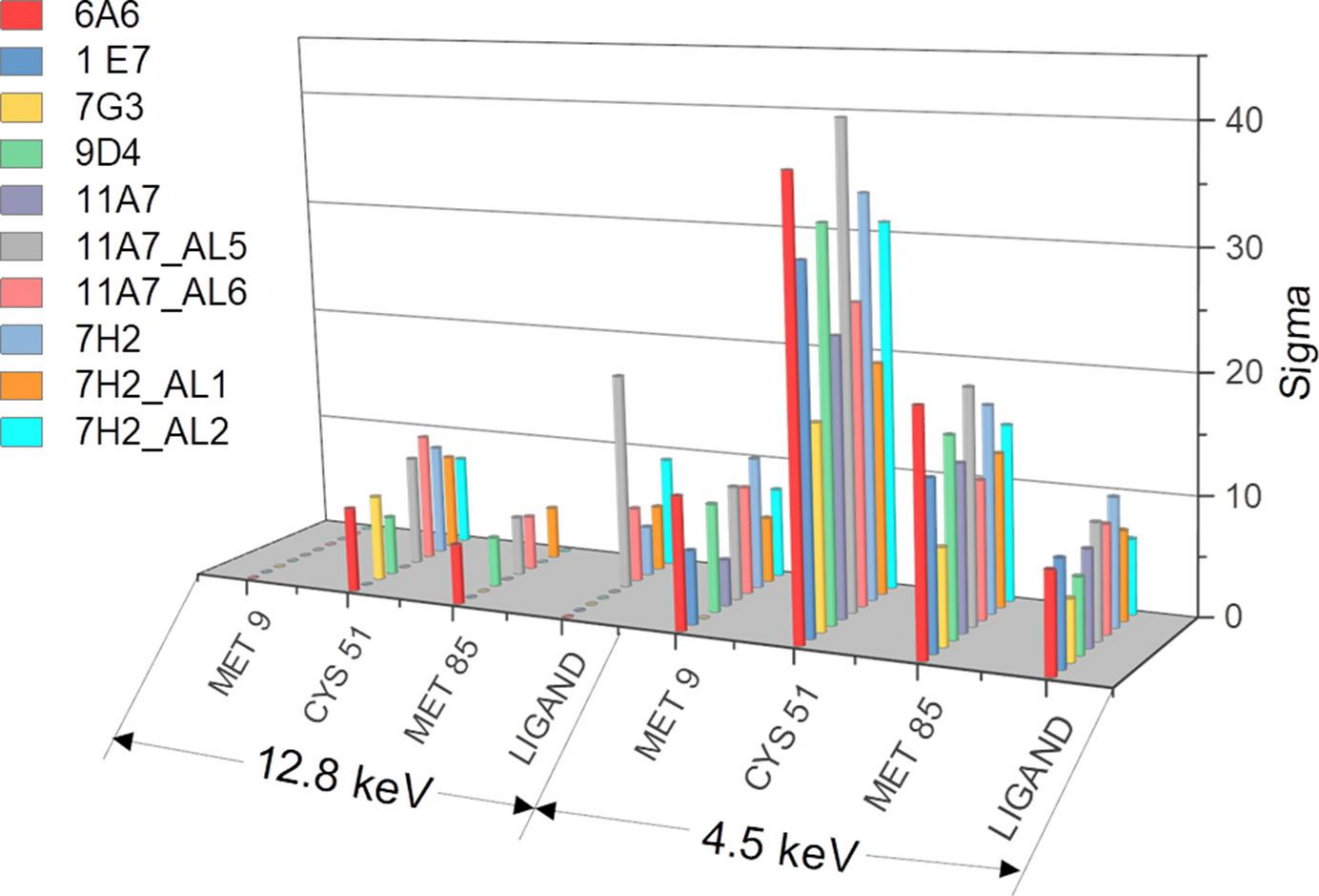
A 3D column chart comparing anomalous peak heights, σ, for S atoms in Met9, Cys51 and Met85 of nsp1 and for S and/or halogen atoms in fragment analogues calculated from the data collected at 12.8 keV compared with those collected at 4.5 keV. If two anomalous signals from fragment analogues appeared (one from S and the other from halogen atoms), only the halogen anomalous peak value was plotted. This chart was prepared in *Origin* 2018 (Moberly *et al.*, 2018 ▸).

To validate the binding of the fragment analogues, we prepared analogue-soaked nsp1 crystals and collected diffraction data at 12.8 keV on MASSIF-1 at ESRF. However, when fitting the fragment analogues into the *mF*_o_ − *DF*_c_ maps, half of the maps showed incomplete fragment density, which is not uncommon for fragments with low binding occupancy. Therefore, we took advantage of the anomalous scattering from the heavy atoms contained in these fragment analogues by calculating anomalous difference Fourier maps from the 12.8 keV data sets using *Dimple* (version 2.6.2; Wojdyr *et al.*, 2013 ▸). The quality of the maps obtained was validated by inspecting the anomalous signal from the S atoms of Met9, Cys51 and Met85 of nsp1 and the sulfur or halogen signals from the fragment analogues in *Coot* (Emsley *et al.*, 2010 ▸; Fig. 2 ▸).

Overall, the anomalous sulfur signal from the cysteine, methionine and sulfur-containing fragment analogues (1E7, 7G3, 6A6, 11A7, 11A7_AL5 and 11A7_AL6) cannot be observed consistently, probably due to the low anomalous contribution to the structure factor *f*′′ (0.2 e) of sulfur at 12.8 keV, which is far from the sulfur absorption edge (Supplementary Fig. S1). Therefore, the anomalous difference Fourier maps calculated from the data sets collected at a standard wavelength (1.0 Å, 12.8 keV) are not sufficient to facilitate the fitting of sulfur-containing fragments. Likewise, the anomalous signal of chlorine could not be observed in the fragment density of 9D4 and only appeared weakly in the density for 7H2 as the *f*′′ of chlorine at 12.8 keV is also low (0.3 e; Supplementary Fig. S1). However, this scenario changed for the bromine or iodine-containing analogues (11A7_AL5, 11A7_AL6, 7H2_AL1 and 7H2_AL2), for which peaks can be observed in the anomalous difference Fourier maps at 12.8 keV because the *f*′′ of iodine and bromine are as high as 3.0 and 0.5 e, respectively, at this beam energy. Site-specific radiation damage was observed in the anomalous difference Fourier map calculated from the 7H2_AL2 data set collected at 12.8 keV, where two adjacent anomalous peaks of 9.2σ and 6.5σ appeared (only the higher peak was plotted in Fig. 2 ▸). This suggests that although anomalous signal from iodine can be observed in the data collected at a standard wavelength, it would still be beneficial to collect data using low doses or at an incident beam energy far from the iodine absorption peak at 5.2 keV to avoid site-specific radiation damage, which will be discussed in more detail below. Additionally, for the quasi-symmetric and planar fragment analogue 7H2_AL1, in which the two substituents are in *para* positions, a single anomalous signal from bromine is adequate to fit the fragment into the density. In contrast, for the asymmetric analogues 11A7_AL5 and 11A7_AL6, a single anomalous signal from iodine or bromine is not sufficient. Therefore, for those analogues that contain both S and Br/I atoms, it is suggested that anomalous data should be collected at lower energy, close to and above the sulfur absorption edge, to obtain the anomalous signals from both heavy atoms in order to unambiguously fit them into the electron density.

To obtain higher quality anomalous difference Fourier maps, nsp1 crystals soaked with the distinct fragment analogues prepared under the same conditions were measured on beamline I23 at DLS at an incident X-ray energy of 4.5 keV. The anomalous signal from sulfur in methionine and cysteine side chains present in nsp1, and from heavy atoms in the fragments, were again visually inspected and the peak heights of these anomalous signals were compared with those extracted from the 12.8 keV data (Fig. 2 ▸). The anomalous signals originating from the S atoms in these residues are visible in all anomalous difference Fourier maps except for that of nsp1–7G3, for which that of the sulfur in Met9 was not observed. This is possibly because of the low *I*/σ(*I*) of this specific data set and the flexibility of Met9 as the first residue at the N-terminus of nsp1. It is clear that the anomalous signals of S atoms from the protein and fragment analogues are significantly stronger and appear consistently in the anomalous difference Fourier maps calculated from the data collected at I23, where the measurements were performed at *E_x_* = 4.5 keV in a vacuum environment to maximize the signal-to-noise ratio (El Omari *et al.*, 2023 ▸). The anomalous signals of halogen atoms in the fragment analogues are also strong. No anomalous peak splitting, which might be caused by site-specific radiation damage, was observed.

### 3.2. Strategy for anomalous scattering data collection and the effects of radiation damage

**Figure 3:**
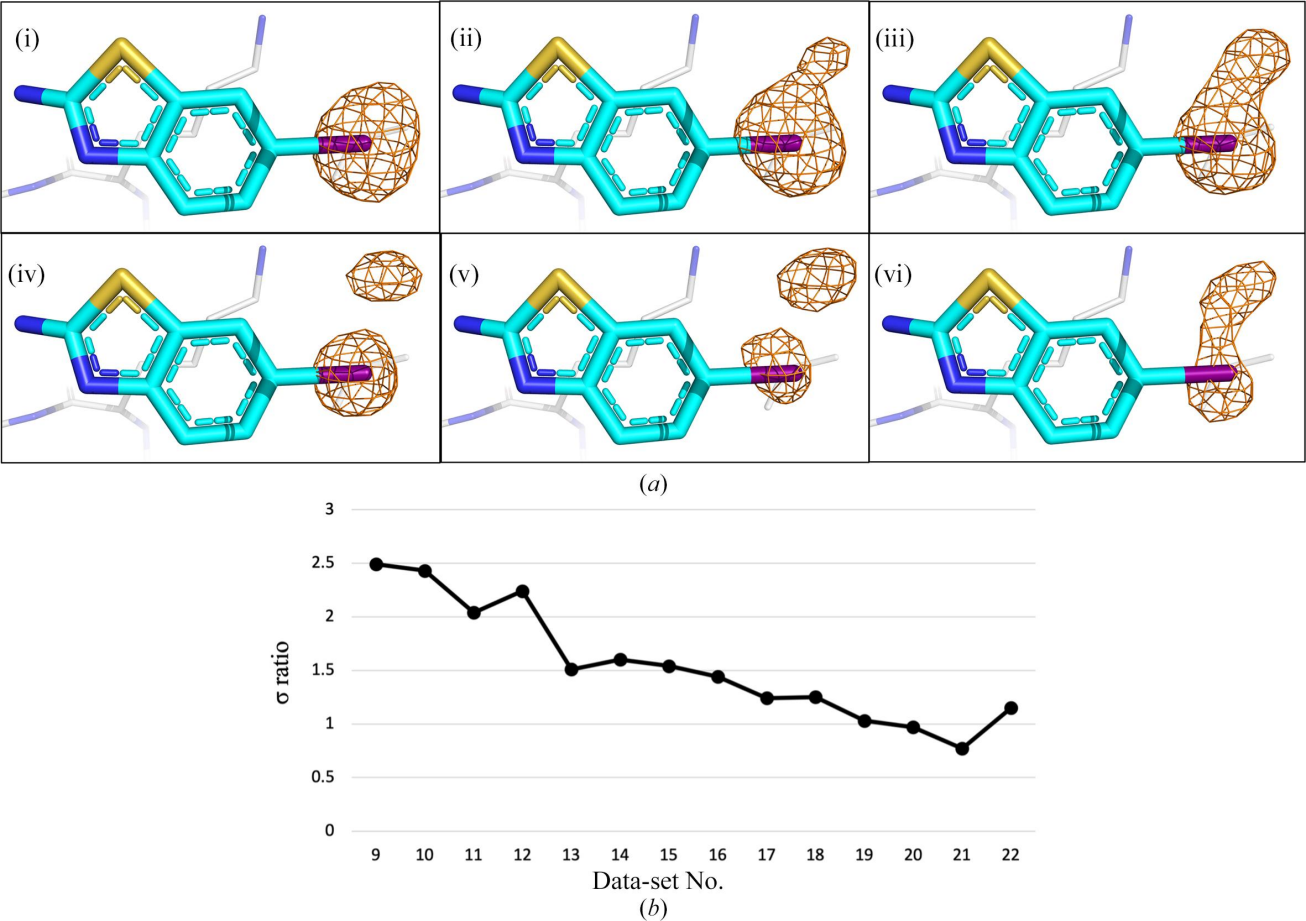
(*a*) Representative transitions of the anomalous difference Fourier maps of the iodine-containing fragment analogue 11A7_AL5 showing site-specific radiation damage that occurs during data collection at *E_x_* = 9 keV. Panels (i)–(vi) show the gradual and continuous development of the second anomalous peak from iodine present in the analogue. For simplicity, only the maps from data sets 8, 9, 13, 17, 20 and 22 are shown. I, N, S and C atoms are coloured purple, blue, yellow and cyan, respectively. The anomalous difference Fourier maps are shaded as an orange mesh (4σ). (*b*) Line graph showing the sigma ratio of iodine anomalous peak heights between the first (initial) and the second (gradually appearing) anomalous peaks. The second peak did not appear in the maps for the first eight data sets, and therefore the graph starts at data set 9.

The wavelength chosen for anomalous data collection on I23 was based on the following considerations. For sulfur- and chloride-containing fragments, 4.5 keV is above their absorption edges, allowing strong anomalous signals to be obtained without compromising resolution (the maximum achievable resolution at 4.5 keV is 1.8 Å due to the I23 beamline detector geometry and the fixed sample-to-detector distance). While the *K* absorption edge of bromine is 13.5 keV, which is beyond the tuneable range, the anomalous contribution to the structure factor from the *L* edge of Br at 4.5 keV (*f*′′ = 3.4 e) is sufficiently high to allow the signal to be confidently observed in the anomalous difference Fourier maps. For iodine-containing fragments, although the *L* absorption edge (5.2 keV) is within the tuneable range, data collection close to and above its absorption edge should be avoided due to the corresponding strong X-ray absorption cross section. As site-specific radiation damage was observed at 12.8 keV, where the *f*′′ of iodine is 3.0 e, we collected data at three incident energies, just below the absorption peak (4.5 keV), above the peak (5.3 keV) and significantly above the peak (9 keV), for both analogues to establish the best approach for data collection for the iodine-containing analogues 7H2_AL2 and 11A7_AL5. By comparing the fragment-binding sites in the three data sets, we observed that a single anomalous peak appeared around the I atom in both the 4.5 and 9 keV maps, while two adjacent anomalous peaks appeared in the 5.3 keV data set for both analogues (Supplementary Fig. S2). This supports our conjecture that site-specific radiation damage is likely to occur due to the strong absorption cross section of iodine at the *L* edge (5.18 keV). Radiation-induced structural changes in proteins are not uncommon, but are a major concern when the measurements are carried out at energies that correspond to the absorption edges of ions, although these changes can be utilized for experimental phasing (Fütterer *et al.*, 2008 ▸; Schiltz *et al.*, 2004 ▸). To test this, we collected 22 data sets at 9 keV, where the absorption of iodine is reduced by an order of magnitude in comparison with the peak value (calculated at https://henke.lbl.gov/optical_constants/atten2.html), from a single crystal soaked with 11A7_AL5. This was conducted to capture the moment of initiation of the site-specific radiation-induced changes. The average dose absorbed by the whole crystal during the collection of one data set at 9 keV was 0.34 MGy, as calculated by *RADDOSE*-3*D* (Bury *et al.*, 2018 ▸). By inspecting the 22 data sets in the order in which they were collected, we observed that the radiation-induced structural changes occurred when the anomalous signal of iodine gradually shifted to the second anomalous peak (starting from the ninth data collection) and that the anomalous signal is redistributed between the two sites until the peak-height ratio reduces to 1. Representative transitions of the anomalous difference Fourier maps are displayed in Fig. 3 ▸(*a*), while the peak-height ratio between the two anomalous signals is plotted in Fig. 3 ▸(*b*) from data set 9. We believe that the absorbed dose could trigger cleavage of the C—I bond, leading to a shift of the I atom away from the C atom to the nearest available space as the absorbed dose increases. The displacement of metal ions induced by radiation has recently been shown in X-ray crystallographic studies of metallo­proteins (Lennartz *et al.*, 2022 ▸), while instances of radiation-induced bond cleavage and the subsequent shift of a Br atom have also been previously documented (Ravelli *et al.*, 2003 ▸). In the case of shifted anomalous signals in the maps from the diffraction data of 11A7_AL5 in complex with nsp1, the peaks are derived from an I atom in two distinct locations. As data collection proceeds, the fraction of cleaved C—I bonds increases, which is manifested as a gradually stronger second anomalous peak in the superposed electron-density maps. A rigorous validation of this hypothesis would require dedicated experiments and theoretical calculations which are outside the scope of this study. Nonetheless, this investigation allowed us to determine a strategy to prevent C—I bond cleavage when using iodine-containing fragments. For anomalous data collection we chose 4.5 keV where, despite being below the *L* absorption edge of iodine, the *f*′′ value (3.4 e) is large enough for iodine to be observed in the anomalous difference Fourier maps. At the same time, the absorption at this energy is relatively low and thus is unlikely to induce radiation damage at typical doses for data collection.

### 3.3. Low-occupancy and planar fragment fitting using anomalous signals and *PanDDA* maps

**Figure 4:**
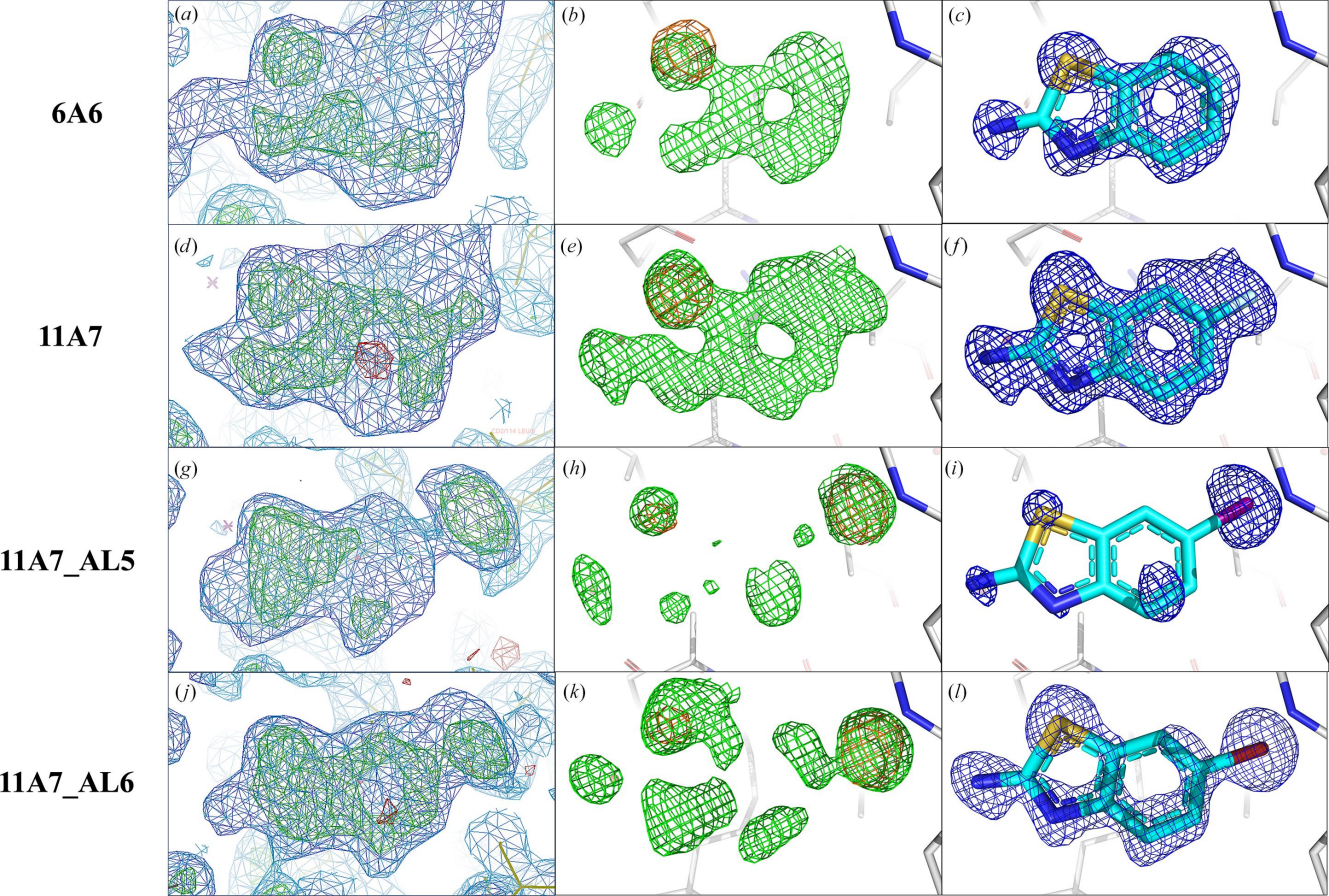
Comparison of various maps in the binding site of analogues 6A6, 11A7, 11A7_AL5 and 11A7_AL6 (row 1 to row 4, respectively). (*a*, *d*, *g*, *j*) *PanDDA* event maps [blue, 1.0σ, background density correction factor (BDC) = 0.37, 0.39, 0.18 and 0.25, respectively] and *Z*-maps (green/red, ±4.0σ). (*b*, *e*, *h*, *k*) Sulfur, iodine and bromine anomalous difference Fourier maps in the fragment region calculated from data collected at 4.5 keV (orange, 4σ) overlaid with *mF*_o_ − *DF*_c_ maps (green, 3.0σ) calculated from the 12.8 keV data. (*c*, *f*, *i*, *l*) Refined 2*mF*_o_ − *DF*_c_ maps (blue, 1.0σ) calculated from the 12.8 keV data with the S, I or Br atoms placed in the centres of their anomalous peaks. The 2*mF*_o_ − *DF*_c_ map is almost complete for 6A6 (*c*), 11A7 (*f*) and 11A7_AL6 (*l*) but is partial for 11A7_AL5 (*i*). In Figs. 4 ▸, 5 ▸ and 6 ▸ and Supplementary Fig. S2, the C, N, O, S, F, Cl, Br and I atoms in the fragments are coloured cyan, blue, red, yellow, light blue, green, dark red and purple, respectively.

Fragment fitting was guided by overlaying anomalous difference Fourier maps generated from data collected at 4.5 keV onto *mF*_o_ − *DF*_c_ maps calculated from the MASSIF-1 data (Figs. 4 ▸, 5 ▸ and 6 ▸). X-ray fluorescence spectra of halogen-containing fragments were also collected to identify chemical ions of interest that might be in the crystal or potentially bind to the protein. The emission spectrum measured at 9.0 keV, as exemplified by the nsp1–11A7_AL5 complex, shows clear peaks assigned to the *K*α lines of sulfur at 2.3 keV and chlorine at 2.6 keV, as well as a peak due to the *L*β line of iodine at 4.3 keV (Supplementary Fig. S3).

A multi-crystal method for extracting weak binding states from conventionally uninterpretable electron density, *PanDDA*, was run on the data collected at 12.8 keV to evaluate its effectiveness in identifying partial occupancy features in the crystallographic data. As expected, *PanDDA* maps show more complete fragment density compared with the standard maps for all fragment analogues. However, the binding orientations and potential alternative orientations of the analogues are still challenging to interpret based solely on *PanDDA* maps (Figs. 4 ▸ *a*, 4 ▸ *d*, 4 ▸ *g*, 4 ▸ *j*, 5 ▸ *a*, 5 ▸ *d*, 5 ▸ *g*, 6 ▸ *a*, 6 ▸ *d* and 6 ▸ *g*).

Among the seven analogues of fragment hit 2E10 (Fig. 1 ▸), 6A6, 11A7, 11A7_AL5 and 11A7_AL6 contain a benzothiazole ring system and an amine substituent at the 2′ position. The only difference between these structures is the substituent at the 6′ position, which is either hydrogen, fluorine, bromine or iodine.

The *PanDDA* map for 6A6 could indicate the location of its single amine substituent in the fragment density, but provides less information on the direction of its ring system due to its quasi-symmetry (Fig. 4 ▸ *a*). The binding orientation is clear when the anomalous signal of sulfur is present (Fig. 4 ▸ *b*). Although the *PanDDA* map for 11A7_AL5 (Fig. 4 ▸ *g*) is interpretable, the *mF*_o_ − *DF*_c_ map (Fig. 4 ▸ *h*) of 11A7_AL5 is at best partial, and both provide little information on binding orientation. Therefore, fitting the fragment analogue into the maps (Fig. 4 ▸ *g* or 4 ▸ *h*) would be challenging. Similarly, through the location of the anomalous peaks from sulfur and iodine and the assignment of the two peaks by comparing the difference in anomalous peak heights between sulfur and iodine (with iodine having a higher anomalous peak height due to its larger *f*′′ value; Fig. 4 ▸ *h*), the binding orientations can be unambiguously determined (Fig. 4 ▸ *i*). The *PanDDA* maps (Figs. 4 ▸ *d* and 4 ▸ *j*) are complete for 11A7 and 11A7_AL6 and are better than the *mF*_o_ − *DF*_c_ maps (Figs. 4 ▸ *e* and 4 ▸ *k*). However, both types of map are sufficient for an experienced crystallographer to manually fit the analogues in the correct orientations (Figs. 4 ▸ *f* and 4 ▸ *l*). Nonetheless, the anomalous difference Fourier map provides further confidence in fitting. Overall, the binding orientations of the four analogues are the same, as expected from their high structural and chemical similarity (Fig. 4 ▸).

**Figure 5:**
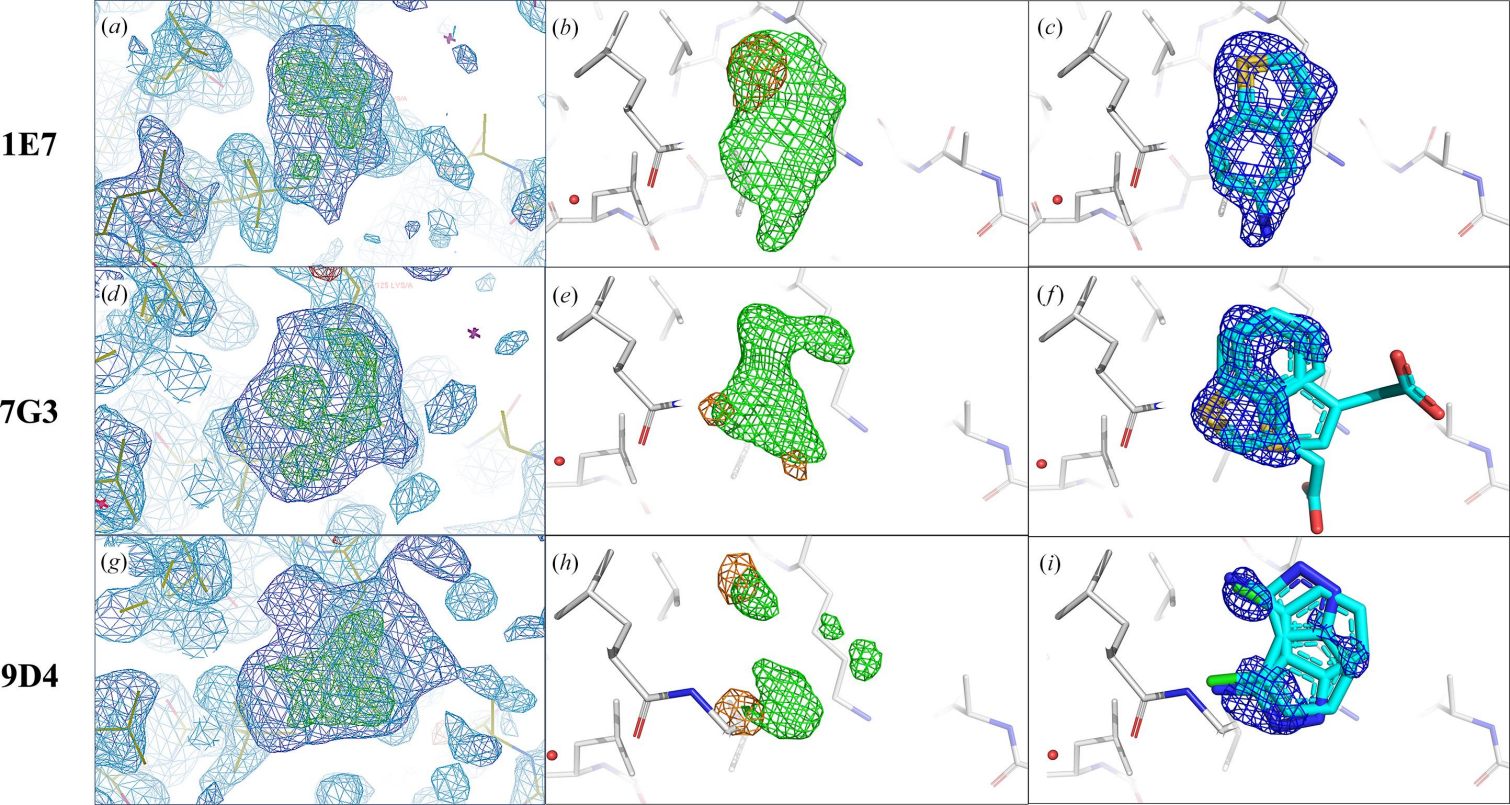
Comparison of various maps in the binding site of analogues 1E7, 7G3 and 9D4 (row 1 to row 3, respectively). (*a*, *d*, *g*) *PanDDA* event maps (BDC = 0.44, 0.27 and 0.21, respectively) and *Z*-maps (green/red, ±4.0σ). (*b*, *e*, *h*) Sulfur and chlorine anomalous difference Fourier maps calculated from data collected at 4.5 keV (orange, 4σ) overlaid with *mF*_o_ − *DF*_c_ maps (green, 3.0σ) calculated from the 12.8 keV data in the fragment region. (*c*, *f*, *i*) Refined 2*mF*_o_ − *DF*_c_ maps (blue, 1.0σ) calculated from the 12.8 keV data with the S or Cl atoms placed in the centres of the anomalous peaks. The electron density completely accounts for 1E7, while only partial density is visible for 7G3 and 9D4, possibly due to their low occupancy. While one binding orientation was identified for 1E7, two binding orientations were evident for both 7G3 and 9D4.

The other three analogues of 2E10, namely 1E7, 7G3 and 9D4 (Fig. 1 ▸), demonstrate more diversity in the five-membered rings fused to the benzene ring. 1E7 and 7G3 share the same ring scaffold, benzothiophene, with a single substituent at distinct positions. For 1E7, an amine is positioned *para* to the sulfur, while a more flexible acetic acid substituent is located in the *meta* position to the sulfur in 7G3, which may explain the missing density for this substituent (Figs. 5 ▸ *d*, 5 ▸ *e* and 5 ▸ *f*). The *mF*_o_ − *DF*_c_ map obtained from a single crystal is as good as the *PanDDA* map of 1E7 (Fig. 5 ▸ *a*), indicating nearly full occupancy and a clear binding orientation (Fig. 5 ▸ *c*). In the anomalous difference Fourier map of 1E7 combined with the *mF*_o_ − *DF*_c_ map, only one sulfur anomalous peak was observed (Fig. 5 ▸ *b*).

In contrast to 1E7, 7G3 represents a fragment analogue that binds with low occupancy, resulting in difficult-to-interpret electron-density maps (Figs. 5 ▸ *d*, 5 ▸ *e* and 5 ▸ *f*). Whereas the *PanDDA* map still covers the core ring structure, it provides no indication of its substituent and binding orientations (Fig. 5 ▸ *d*). Interestingly, even at a resolution of 1.2 Å the *mF*_o_ − *DF*_c_ map of 7G3 is only partially visible (Fig. 5 ▸ *e*), and it is difficult to fit it confidently into the density. To complicate matters, two peaks were observed for the S atom in 7G3 (Fig. 5 ▸ *e*). In such a challenging case, the anomalous difference Fourier map allowed confident fragment fitting, exemplifying how useful anomalous signals can be when working with low-occupancy fragments that bind in two distinct orientations.

Although maintaining a fused two-ring core, 9D4 has an indazole ring system and two substituents, one on each ring. An amine substituent is present at the 3′ position, while a chloro substituent is at the 4′ position (Fig. 1 ▸). For this fragment analogue, the *mF*_o_ − *DF*_c_ map is uninterpretable (Fig. 5 ▸ *h*). The *PanDDA* map nearly covers the core ring system of 9D4 and again highlights the strength of this pan-data-set approach, but it provides limited information about the position of its substituent and potential binding orientations (Fig. 5 ▸ *g*). Facilitated by the anomalous difference Fourier map, two binding orientations were clearly suggested by chlorine anomalous peaks in the density (Figs. 5 ▸ *h* and 5 ▸ *i*).

**Figure 6:**
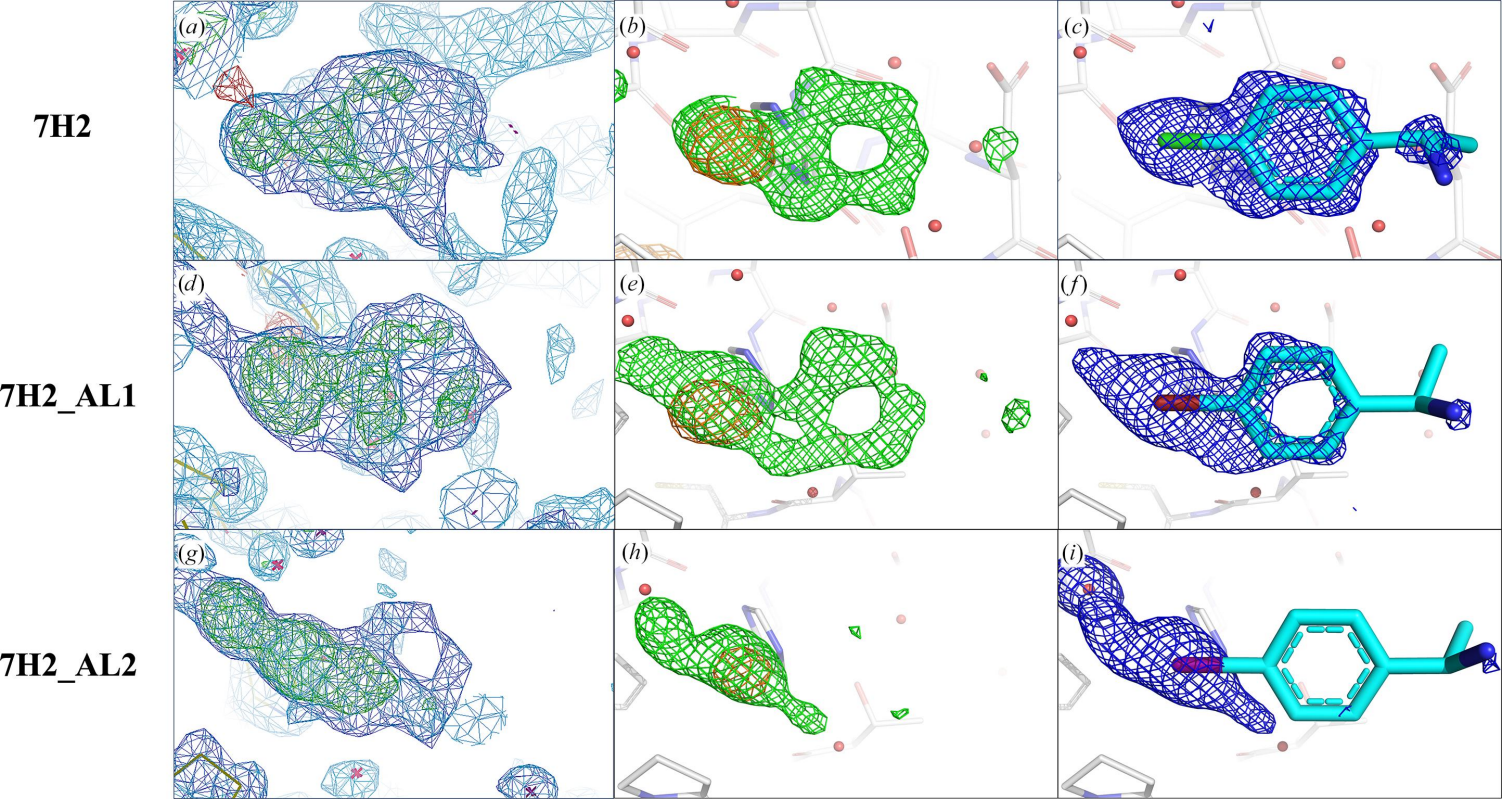
Comparison of various maps in the binding site of analogues 7H2, 7H2_AL1 and 7H2_AL2 (row 1 to row 3, respectively). (*a*, *d*, *g*) *PanDDA* event maps (BDC = 0.34, 0.33 and 0.48, respectively) and *Z*-maps (green/red, ±4.0σ). (*b*, *e*, *h*) Chlorine, bromine and iodine anomalous difference Fourier maps calculated from data collected at 4.5 keV (orange, 4σ) overlaid with *mF*_o_ − *DF*_c_ maps (green, 3.0σ) calculated from the 12.8 keV data in the fragment region. (*c*, *f*, *i*) Refined 2*mF*_o_ − *DF*_c_ maps (blue, 1.0σ) of 7H2, 7H2_AL1 and 7H2_AL2 calculated from the 12.8 keV data with the Cl, Br or I atom placed in the centre of the anomalous peaks. The electron density mostly accounts for 7H2, while for the two analogues the density systematically degrades, possibly due to their low occupancy.

7H2_AL1 and 7H2_AL2 are two analogues of the previously reported fragment hit 7H2 (Ma *et al.*, 2022 ▸) in which the chlorine substituent is replaced with bromine and iodine (Fig. 1 ▸), respectively. 7H2 displays reasonable *PanDDA* and *mF*_o_ − *DF*_c_ maps; however, the direction of its two substituents was ambiguous. The combination of the anomalous difference Fourier map and the *mF*_o_ − *DF*_c_ map allowed unambiguous fitting of the fragment (Ma *et al.*, 2023 ▸; Figs. 6 ▸ *b* and 6 ▸ *c*). Similarly, the nearly complete *PanDDA* (Fig. 6 ▸ *d*) and partial *mF*_o_ − *DF*_c_ maps (Fig. 6 ▸ *e*) of 7H2_AL1 present two possible binding orientations. By locating the anomalous signal from bromine, an unambiguous binding orientation of 7H2_AL1 can be determined (Figs. 6 ▸ *e* and 6 ▸ *f*). However, for 7H2_AL2 the density is clearly compromised and distorted by site-specific radiation damage in the *mF*_o_ − *DF*_c_ map (Fig. 6 ▸ *h*). By locating the anomalous signal of the iodine substituent, the fragment can still be confidently placed into the remaining density, in particular in the *PanDDA* map. The three analogues share the same binding orientation, with the halogen atoms pointing towards the protein to anchor the fragments in the binding pocket (Figs. 6 ▸ *c*, 6 ▸ *f* and 6 ▸ *i*).

## 4. Discussion

FBDD has emerged as a powerful strategy for developing novel lead compounds and advancing drug development. However, FBDD also presents challenges that differentiate it from traditional small-molecule drug-discovery approaches. A fundamental aspect is the markedly weak binding affinity of fragments for their targets, which is typically in the low-millimolar range. Additionally, the fragments are small, simple and typically incorporate at least one aromatic ring, therefore having fewer rotatable bonds, which renders them planar and quasi-symmetric. These intrinsic features introduce additional complexities in determining their binding orientations.

This challenge can be addressed by implementing the *PanDDA* approach, which identifies binding events by comparing data sets from crystals soaked with fragments with native data sets, allowing the identification of fragments with a statistically reliable degree of confidence (Pearce *et al.*, 2015 ▸). However, the fragment density in *PanDDA* maps for low-occupancy binding events may still be insufficient to ascertain the binding orientation(s) of hits due to their inherent features, such as small size and planarity. To overcome this issue, the combination of *PanDDA* maps with anomalous signals generated from atoms in fragment analogues not only confirms the binding orientation but can also suggest the presence of multiple binding orientations. The occupancy of each orientation for the same fragment analogue can also be estimated from the ratio of anomalous peak heights. Consequently, these two methods are complementary, and when employed in conjunction they can offer unequivocal information about fragment binding conformations.

**Figure 7:**
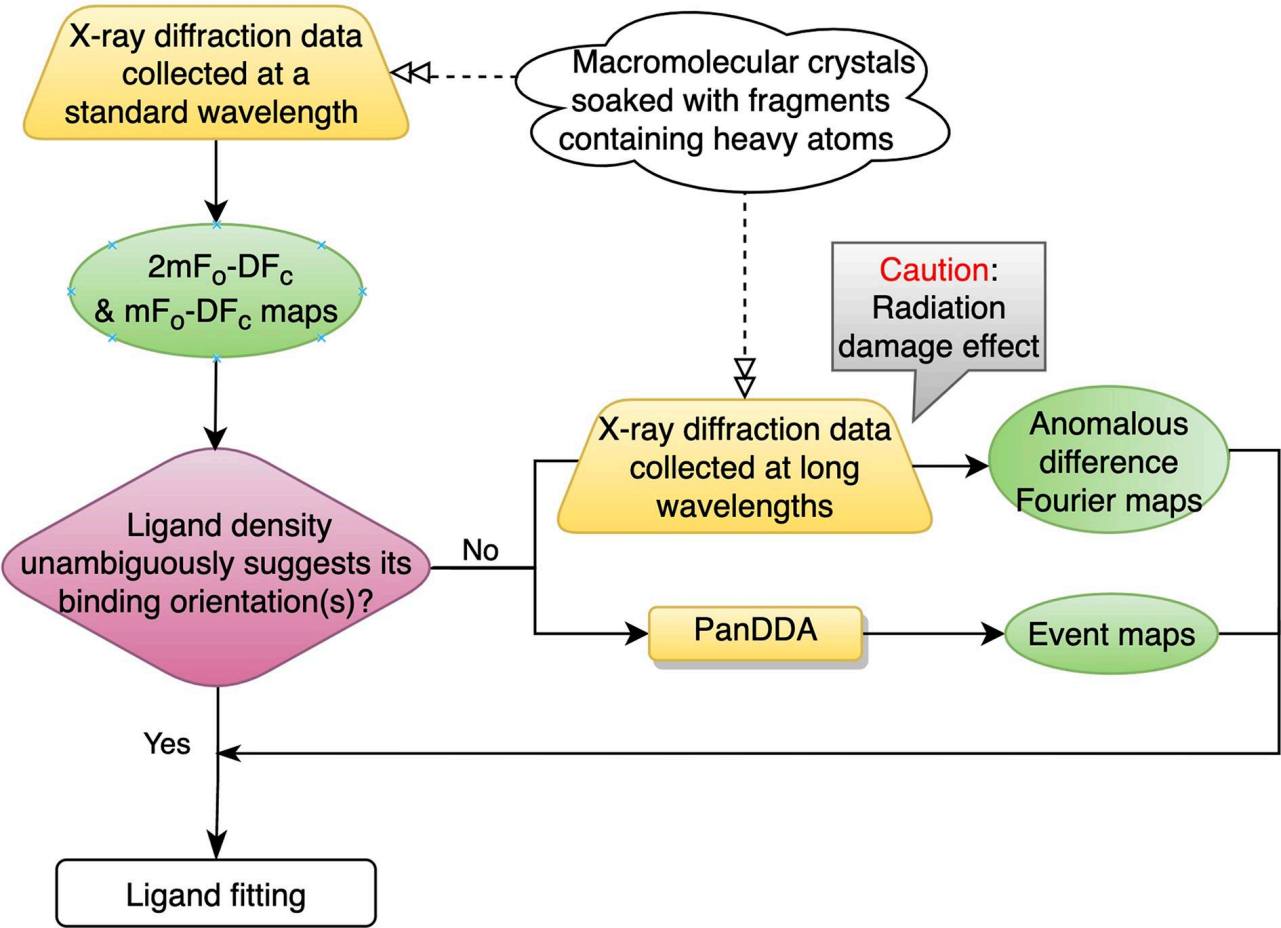
Schematic workflow for correctly placing low-occupancy fragments into incomplete electron density during FBDD.

This study has the potential to inform good practice for the problem of correctly placing low-occupancy fragments into incomplete electron density in FBDD. The method is well suited to determine the binding orientations of fragments. A schematic summary of the general workflow is provided in Fig. 7 ▸. X-ray diffraction data are first collected at a standard wavelength from crystals soaked with fragments. Fourier maps are generated in the single-crystal system, where well defined ligand density can unambiguously guide ligand fitting. Challenging-to-fit low-occupancy fragments, showing partial or uninterpretable fragment density, are then selected for long-wavelength experiments with careful design of the data-collection parameters, considering potential radiation-damage effects caused by the absorbed dose. Anomalous difference Fourier maps are then calculated from the long-wavelength data, and event maps are computed by *PanDDA* in the multi-crystal system. Determining the binding orientation(s) of low-occupancy fragments is achieved by considering the complementary information from both *PanDDA* event maps and anomalous difference Fourier maps.

Radiation-damage effects in data collection for fragments containing bromine and iodine have rarely been considered. Anomalous data have also rarely been applied to simultaneously determine the binding modes of fragments containing S and halogen atoms. This study of data-collection strategies for fragments containing S and/or halogenated atoms or substituents suggest that a carefully designed strategy for data collection is necessary depending on the purpose of the study. For the identification of fragment hits containing iodine, a low dose during data collection is recommended to avoid site-specific radiation damage, in particular when the X-ray energy is close to the iodine absorption edge. For the determination of binding orientations of fragments containing both S/Cl and halogen atoms, the incident X-ray energy should be above and close to the sulfur/chlorine absorption edge to ensure that the anomalous signals of both can be observed for unambiguous manual fragment placement.

## 5. Conclusion

In conclusion, this study has shown how the challenge of determining the binding orientation(s) for low-occupancy and difficult-to-fit fragments can successfully be addressed for sulfur- and/or halogen-containing fragments. This method is poised to significantly improve the efficacy and success rate of FBDD during rational drug design.

## Supplementary Material

PDB reference: nsp1–1E7, 8rf2

PDB reference: nsp1–7G3, 8rf3

PDB reference: nsp1–9D4, 8rf4

PDB reference: nsp1–11A7, 8rf5

PDB reference: nsp1–11A7_AL5, 8rf6

PDB reference: nsp1–11A7_AL6, 8rf8

PDB reference: nsp1–7H2_AL1, 8rfc

PDB reference: nsp1–7H2_AL2, 8rfd

PDB reference: nsp1–6A6, 8rff

Supplementary Table and Figures. DOI: 10.1107/S2059798324004480/gm5104sup1.pdf

## Figures and Tables

**Table:** Values in parentheses are for the highest resolution shell.

	1E7	7G3	9D4	6A6	11A7
Wavelength (Å)	2.8/1.0	2.8/1.0	2.8/1.0	2.8/1.0	2.8/1.0
Resolution range (Å)	140.20–1.80 (1.83–1.80)/35.20–1.44 (1.46–1.44)	35.60–1.80 (1.83–1.80)/35.62–1.23 (1.25–1.23)	35.59–1.77 (1.80–1.77)/35.76–1.11 (1.13–1.11)	140.50–1.80 (1.83–1.80)/35.46–1.31 (1.33–1.31)	140.52–1.80 (1.83–1.80)/35.65–1.10 (1.12–1.10)
Space group	*P*4_3_2_1_2	*P*4_3_2_1_2	*P*4_3_2_1_2	*P*4_3_2_1_2	*P*4_3_2_1_2
*a*, *b*, *c* (Å)	36.5, 36.5, 140.2/36.4, 36.4, 140.2	36.8, 36.8, 140.6/36.8, 36.8, 140.8	36.8, 36.8, 140.8/37.0, 37.0, 141.5	36.8, 36.8, 140.5/36.6, 36.6, 140.7	36.7, 36.7, 140.5/36.8, 36.8 140.9
α, β, γ (°)	90, 90, 90/90, 90, 90	90, 90, 90/90, 90, 90	90, 90, 90/90, 90, 90	90, 90, 90/90, 90, 90	90, 90, 90/90, 90, 90
Total reflections	129747 (2810)/428587 (18947)	104736 (2014)/718205 (34980)	110482 (1768)/924139 (35815)	263258 (5603)/222080 (9736)	122663 (2695)/951652 (39918)
Unique reflections	9408 (455)/18158 (826)	8999 (404)/29312 (1417)	9161 (340)/38471 (1831)	8044 (343)/24079 (1155)	8084 (340)/40695 (2017)
Multiplicity	13.8 (6.2)/23.6 (22.9)	11.6 (5.0)/24.5 (24.7)	12.1 (5.2)/24.0 (19.6)	32.7 (16.3)/9.2 (8.4)	15.2 (7.9)/23.4 (19.8)
Completeness (%)	99.3 (95.8)/99.7 (94.4)	93.0 (83.8)/99.4 (100.0)	90.6 (71.7)/95.4 (92.6)	83.3 (70.9)/100.0 (100.0)	84.2 (73.0)/9.6 (100.0)
Anomalous completeness (%)	99.2 (96.7)/99.7 (94.3)	90.5 (76.1)/99.5 (100.0)	92.5 (74.8)/96.7 (93.8)	84.4 (72.3)/99.6 (99.8)	85.2 (73.1)/99.6 (100.0)
Mean *I*/σ(*I*)	23.5 (2.0)/11.8 (1.2)	16.1 (0.6)/10.1 (1.0)	29.0 (4.9)/10.3 (0.5)	46.0 (9.4)/17.8 (2.4)	32.8 (9.7)/7.2 (1.1)
*R*_meas_ (%)	5.9 (25.1)/15.4 (174.8)	7.8 (187.1)/16.6 (569.4)	5.1 (30.3)/15.6 (624.6)	6.2 (15.2)/6.1 (78.6)	8.3 (31.6)/27.3 (395.1)
CC_1/2_ (%)	100.0 (96.2)/99.1 (73.2)	99.9 (35.1)/68.5 (38.1)	99.9 (93.3)/99.4 (39.3)	99.9 (98.4)/99.9 (81.4)	99.8 (92.2)/98.8 (39.4)

**Table:** 

	11A7_AL5	11A7_AL6	7H2_AL1	7H2_AL2
Wavelength (Å)	2.8/1.0	2.8/1.0	2.8/1.0	2.8/1.0
Resolution range (Å)	140.75–1.80 (1.83–1.80)/35.60–1.08 (1.10–1.08)	36.87–1.77 (1.80–1.77)/35.64–1.13 (1.14–1.13)	141.40–1.80 (1.83–1.80)/35.65–1.10 (1.12–1.10)	140.65–1.80 (1.83–1.80)/35.65–1.13 (1.15–1.13)
Space group	*P*4_3_2_1_2	*P*4_3_2_1_2	*P*4_3_2_1_2	*P*4_3_2_1_2
*a*, *b*, *c* (Å)	36.6, 36.6, 140.8/36.8, 36.8, 141.0	36.9, 36.9, 141.2/36.8, 36.8, 140.9	36.8, 36.8, 141.4/36.8, 36.8, 142.1	36.7, 36.7, 140.7/36.8, 36.8 141.4
α, β, γ (°)	90, 90, 90/90, 90, 90	90, 90, 90/90, 90, 90	90, 90, 90/90, 90, 90	90, 90, 90/90, 90, 90
Total reflections	262680 (5235)/1001686 (39315)	110085 (1687)/809556 (33631)	257401 (5268)/862099 (33096)	67618 (1419)/639742 (25604)
Unique reflections	9665 (476)/42597 (2026)	9510 (361)/37308 (1866)	9794 (471)/41291 (2034)	9513 (458)/37218 (1840)
Multiplicity	27.2 (11.0)/23.5 (19.4)	11.6 (4.7)/21.7 (18.0)	26.3 (11.2)/20.9 (16.3)	7.1 (3.1)/17.2 (13.9)
Completeness (%)	100.0 (99.0)/99.7 (98.9)	93.4 (74.0)/98.0 (100.0)	100.0 (97.5)/99.6 (100.0)	98.8 (95.2)/99.5 (100.0)
Anomalous completeness (%)	99.9 (98.7)/99.9 (99.5)	90.7 (70.9)/97.5 (100.0)	99.8 (96.8)/99.7 (100.0)	98.6 (92.7)/99.6 (100.0)
Mean *I*/σ(*I*)	47.0 (13.8)/10.3 (1.6)	24.6 (7.9)/12.1 (0.8)	12.3 (1.1)/12.0 (0.9)	23.3 (5.7)/11.3 (1.2)
*R*_meas_ (%)	6.5 (14.5)/10.5 (374.9)	8.0 (17.6)/8.6 (313.8)	13.4 (35.9)/12.4 (394.4)	6.6 (20.9)/11.3 (247.0)
CC_1/2_ (%)	99.9 (99.4)/99.8 (48.3)	99.7 (97.1)/99.7 (53.8)	99.8 (93.4)/99.6 (41.9)	99.6 (93.7)/99.7 (46.4)

**Table 2:** Refinement statistics for nsp1–fragment complexes The structure factors used to generate the nsp1–fragment complexes were from high-resolution data collected on MASSIF-1 at 12.8 keV. Values in parentheses are for the highest resolution shell.

	1E7	7G3	9D4	6A6	11A7	11A7_AL5	11A7_AL6	7H2_AL1	7H2_AL2
PDB code	8rf2	8rf3	8rf4	8rff	8rf5	8rf6	8rf8	8rfc	8rfd
*R*_cryst_/*R*_free_ (%)	16.61 (23.06)/22.84 (31.61)	17.60 (31.79)/21.99 (32.14)	18.85 (37.04)/21.40 (35.46)	18.17 (24.83)/20.89 (31.48)	17.26 (31.76)/20.09 (30.93)	18.53 (30.84)/21.49 (34.99)	19.47 (35.65)/24.44 (39.18)	18.31 (32.75)/19.59 (40.87)	17.51 (27.80)/19.91 (32.08)
Reflections used in refinement	17987 (1734)	29028 (2800)	37566 (3094)	24078 (2341)	40470 (3941)	42553 (4093)	37093 (3705)	40779 (3958)	37212 (3632)
Reflections used for *R*_free_	884 (72)	1467 (126)	1876 (160)	1158 (107)	2034 (215)	2118 (189)	1891 (202)	2058 (210)	1850 (170)
No. of non-H atoms									
Total	1035	1044	1022	1029	1046	1037	1036	1037	1021
Protein	956	936	923	930	945	944	943	942	931
Ligand	10	26	22	10	11	16	11	10	10
Solvent	69	82	77	89	90	82	82	85	80
Average *B* factors (Å^2^)									
Overall	33.57	26.85	26.60	21.42	23.88	25.24	25.33	24.31	27.44
Protein	32.88	26.41	25.89	20.80	22.74	24.86	24.84	23.67	26.36
Ligands	38.92	29.92	38.46	21.82	28.20	17.15	18.44	21.88	40.61
Solvent	42.36	30.93	31.64	27.90	35.33	30.73	31.79	31.64	38.43
Ligand occupancy	0.75	0.36/0.27	0.40/0.19	0.63	0.81	0.23	0.35	0.36	0.28
Wilson *B* factor (Å^2^)	23.73	17.18	16.53	16.34	15.22	15.48	16.07	15.26	16.06
R.m.s.d., bond lengths (Å)	0.010	0.010	0.012	0.013	0.010	0.014	0.012	0.013	0.011
R.m.s.d, angles (°)	1.19	1.43	1.53	1.73	1.22	1.55	1.56	1.61	1.49
Ramachandran statistics									
Favoured (%)	99.14	96.55	98.28	99.14	98.28	98.28	98.28	100.0	97.41
Allowed (%)	0.86	3.45	1.72	0.86	1.72	1.72	1.72	0.00	2.59
Outliers (%)	0.00	0.00	0.00	0.00	0.00	0.00	0.00	0.00	0.00
Rotamer outliers (%)	1.90	0.97	3.00	5.88	0.95	0.00	1.96	0.96	2.94
Clashscore	2.55	1.04	2.11	3.71	2.59	7.30	4.15	2.09	1.06
