# Supplementary material for: High-confidence placement of low-occupancy fragments into electron density using the anomalous signal of sulfur and halogen atoms
Source: Acta Crystallogr D Struct Biol. 2024 Jun 5;80(Pt 6):451–63. doi: 10.1107/S2059798324004480 (PMC11154595; doi:10.1107/S2059798324004480)
Supplement: Supplementary file 1 [file d-80-00451-sup1.pdf]

STRUCTURAL BIOLOGY

Volume 80 (2024)

Supporting information for article:

High-confidence placement of low-occupancy fragments into electron density using the anomalous signal of sulfur and halogen atoms

Shumeng Ma, Shymaa Damfo, Matthew W. Bowler, Vitaliy Mykhaylyk and Frank Kozielski

Table S1 SMILES codes of fragment analogues and their parental fragment hits.

Fragments	SMILES codes
1E7	NC1=CC(C=CS2)=C2C=C1
2E10	O=C(C)NC1=CC(CCC2)=C2C=C1
7G3	O=C(O)CC1=CSC2=C1C=CC=C2
6A6	NC1=NC(C=CC=C2)=C2S1
9D4	NC1=NNC2=C1C(Cl)=CC=C2
11A7	NC1=NC(C=CC(F)=C2)=C2S1
11A7_AL5	NC1=NC2=CC=C(C=C2S1)I
11A7_AL6	NC1=NC2=CC=C(C=C2S1)Br
7H2	N[C@@H](C)C(C=C1)=CC=C1Cl
7H2_AL1	C[C@H](C1=CC=C(Br)C=C1)N
7H2_AL2	C[C@H](C1=CC=C(I)C=C1)N

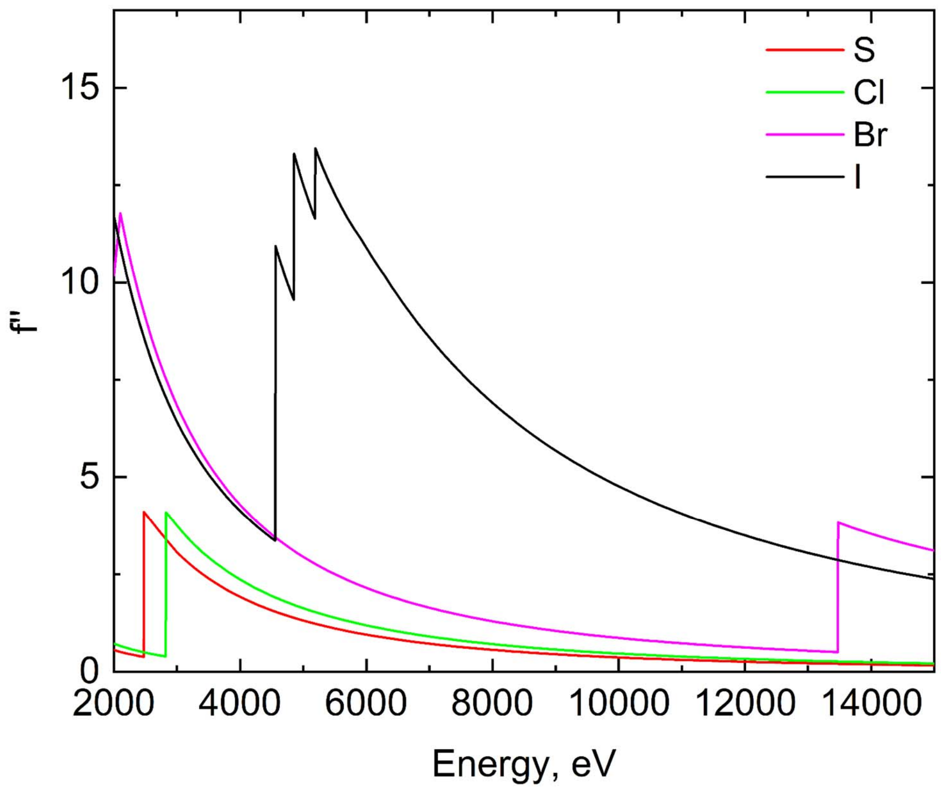

Figure S1 Energy dependence of the anomalous contribution to the structural factor f'' for ions monitored in this study (data from www.bmsc.washington.edu). The value of f'' manifests a sharp increase at the K-edge of S and Cl or L-edges of Br and I, enabling the identification of the ion position.

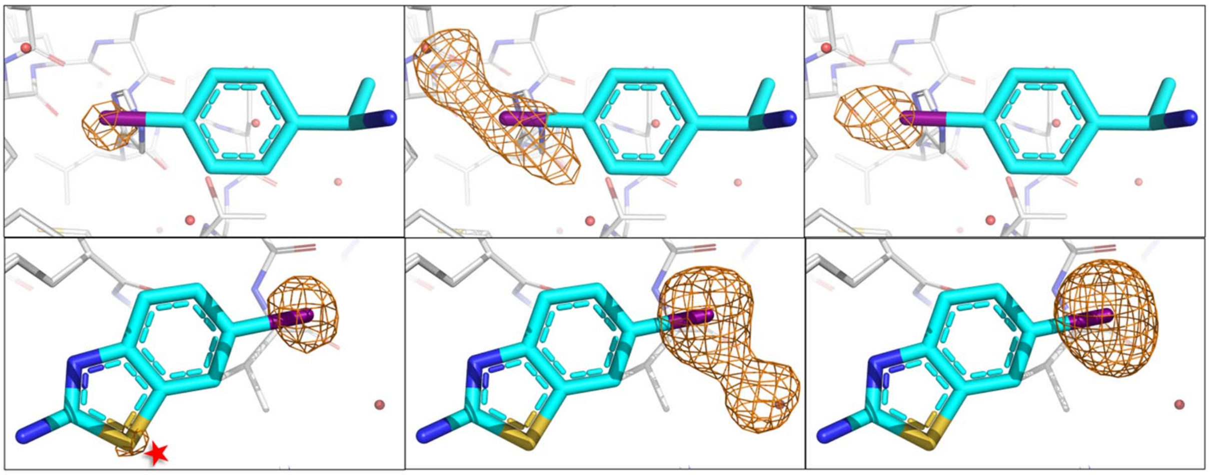

Figure S2 Differences in anomalous difference Fourier maps of iodine-containing fragment analogues 7H2_AL2 **(A-C)** and 11A7_AL5 **(D-F)** collected at incident X-ray energies of 4.5 keV (**A** and **D**), 5.3 keV (**B** and **E**) and 9.0 keV (**C** and **F**). The anomalous difference Fourier maps (contoured at 4 σ level) are shown in orange. Anomalous peaks from the sulphur in 11A7_AL5 are only visible in (**D**) for the 4.5 keV map (indicated by a red star) due to its low f'' value at other two energies.

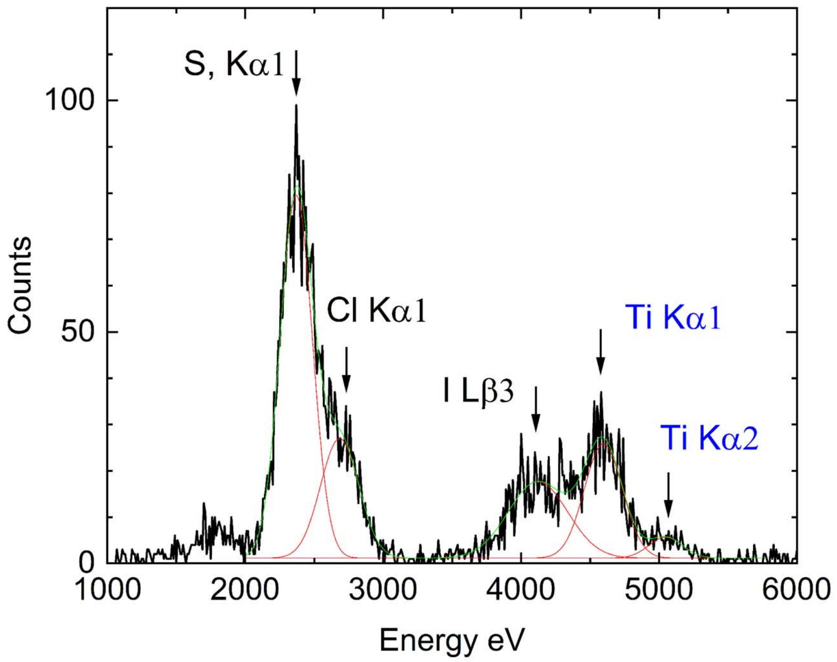

Figure S3 X-ray fluorescence spectrum of the nsp1-11A7_AL5 complex measured at 9 keV beam energy. The Ti K-lines are artefacts caused by a kapton cryo-loop which contained an admixture of titanium.
